# Plasma Homocysteine Concentrations in Horses with Left-Sided Valvular Heart Disease

**DOI:** 10.3390/ani16152307

**Published:** 2026-07-26

**Authors:** Patricia Egli, Elizabeth Williams Louie, Martina Stirn, Gunther van Loon, Annelies Decloedt, Colin C. Schwarzwald, Katharyn J. Mitchell

**Affiliations:** 1 Equine Department, Vetsuisse Faculty, University of Zurich, 8057 Zurich, Switzerland; patricia.egli@uzh.ch (P.E.); cschwarzwald@vetclinics.uzh.ch (C.C.S.); 2 Department of Clinical Sciences, College of Veterinary Medicine, Cornell University, Ithaca, NY 14853, USA; egw43@cornell.edu; 3 Clinical Laboratory, Vetsuisse Faculty, University of Zurich, 8057 Zurich, Switzerland; 4 Equine Cardioteam Ghent, Department of Large Animal Internal Medicine, Faculty of Veterinary Medicine, Ghent University, 9820 Merelbeke, Belgium

**Keywords:** cardiac biomarker, equine, heart, hyperhomocysteinemia, regurgitation

## Abstract

In human medicine, homocysteine, a sulfur-containing amino acid, is used as a biomarker reflecting endothelial dysfunction, inflammation, thrombogenesis, oxidative stress and cardiovascular disease. An automated enzyme-cycling assay for homocysteine, established for humans, has been validated and reference ranges have been created for horses. In this study the homocysteine assay was further evaluated by assessing short-term stability (whole blood and plasma samples stored at 4 °C and room temperature for 24 h), long-term stability (two years), the effect of different anticoagulants (lithium heparin, sodium citrate, EDTA, no additives) and repeated freeze–thaw cycles (five times). Further, associations between plasma homocysteine concentrations ([HCY]_p_) and echocardiographic variables of left heart size, left heart function, and left-sided valve regurgitation were investigated. Homocysteine concentrations remained stable in whole blood at room temperature for at least four hours, or longer when plasma is separated and kept at 4 °C. Samples could be collected in serum blood tubes, lithium heparin (LH), or EDTA without affecting homocysteine concentrations ([HCY]). Plasma [HCY] in frozen samples increased slightly with storage time (years), though up to five freeze–thaw cycles did not affect [HCY]_p_. In a cohort of 169 horses with left-sided valvular regurgitation, plasma creatinine concentrations and age were positively associated with higher [HCY]_p_, but no association between [HCY]_p_ and changes in cardiac size, function or severity of valvular regurgitation could be established. Homocysteine is not associated with left-sided valvular heart disease in horses and is not a useful biomarker for structural or functional cardiac changes.

## Simple Summary

Homocysteine, a sulfur-containing amino acid, is used as a biomarker reflecting endothelial dysfunction, inflammation, thrombogenesis, and oxidative stress. In humans there is an established association between hyperhomocysteinemia and cardiovascular disease. An automated enzyme-cycling assay has been validated and reference intervals for horses have been established, but associations between homocysteine and cardiovascular disease remain under investigation. The aim of this study was to investigate the effect of sample handling and storage conditions using the established homocysteine assay with equine samples, and to measure plasma homocysteine concentrations in horses with left-sided valvular heart disease. Blood samples can be collected in lithium heparin, EDTA, or serum blood tubes, and homocysteine concentrations remain stable for at least four hours regardless of sample handling. Furthermore, while plasma homocysteine concentrations increased slightly during storage over time, samples can undergo up to five freeze–thaw cycles, making this test feasible in a practical setting. Plasma homocysteine concentrations were positively correlated with plasma creatinine concentrations and age but were not associated with echocardiographic indices of left atrial and ventricular size and function or severity of valvular regurgitation. Measurement of homocysteine as a risk factor for and biomarker of cardiovascular disease appears less useful in horses than in people.

## 1. Introduction

Homocysteine (HCY) is a sulfur-containing amino acid synthesized as an intermediate product from methionine, an essential amino acid [1,2]. Vitamin B_12_ and folic acid are essential cofactors for the remethylation of homocysteine to methionine, whereas vitamin B_6_ acts as a cofactor in the transsulfuration pathway converting HCY to cysteine. Deficiencies of these cofactors (e.g., Vit B_12_ deficiency caused by dietary factors, malabsorption or medications like proton pump inhibitors) impair the HCY metabolism and result in accumulation of HCY in the blood [1,2,3,4,5]. Further, genetic mutations resulting in abnormal cysteine and methionine metabolism or chronic kidney disease also result in elevated blood HCY concentrations ([HCY]) [2,6]. Hyperhomocysteinemia (HHCY) alters endothelial and smooth muscle cell function, causing increased formation of reactive oxygen species and alterations in coagulation profiles, increasing the risk for atherosclerotic cardiovascular disease, stroke, peripheral arterial occlusive disease, and venous thrombosis in humans [7,8]. Due to its wide range of functions, HCY has also been investigated in humans in relation to vascular diseases, as well as neurological disorders [9], dementia [10], thrombosis [11], hypothyroidism [12], and age-associated disorders [13].

In people, HHCY is an established risk factor associated with cardiovascular disease [14,15,16,17,18]. Hyperhomocysteinemia has been associated with structural heart disease, including increased left ventricular mass and dilated cardiomyopathy [14], left ventricular hypertrophy in adult humans with chronic kidney disease [19], and atrial fibrillation [20,21]. Hyperhomocysteinemia has not only been investigated as a diagnostic biomarker, but also as an etiological factor, and lowering plasma homocysteine concentrations ([HCY]_p_) may reduce cardiovascular disease risk [17].

In horses, atrial and ventricular remodeling are important clinical findings, often associated with valvular heart disease, which can be identified using echocardiography [22]. Frequently occurring secondary to left-sided valvular heart disease (mitral or aortic regurgitation), left cardiac chamber enlargement can have important sequelae. Enlargement of the atria increases the risk of developing atrial fibrillation (AF), a common, at times performance limiting arrhythmia in horses (affecting 2.3–4.9% of the horse population) [23,24,25]. Ventricular enlargement can predispose to ventricular arrhythmias that can potentially be malignant and dangerous for horse and rider [26,27]. Clinicians are regularly asked to provide recommendations whether horses with left-sided valvular heart disease are safe to ride or not. Such assessments can be challenging and vary amongst clinicians [26]. Blood biomarkers that accurately identify clinically relevant valvular heart disease in horses would be helpful diagnostic tools to signify that further investigation, such as echocardiography, is indicated. In veterinary medicine, the usefulness of measuring [HCY]_p_ as a prognostic or diagnostic biomarker has only rarely been investigated [28,29,30,31,32,33,34,35]. A commercially available assay has been validated for the use in horses, and reference intervals for [HCY]_p_ in healthy horses have been created [28]. Plasma [HCY] of horses with AF were not different from healthy horses [28]. However, horses with AF do not always have clear macrostructural myocardial changes and there is still a large gap in understanding the potential usefulness of [HCY]_p_ as a diagnostic marker of health and disease in horses [26,30,36]. In an unpublished pilot study performed by the authors, horses with the highest [HCY]_p_ had left-sided valvular regurgitation, with evidence of atrial and ventricular remodeling, suggesting this as an interesting avenue for further investigation.

The objectives of this study were to investigate the effect of sample handling, processing and storage on [HCY] in equine samples, measured with the automated enzyme-cycling assay (Roche Cobas HCYS Homocysteine Enzymatic Assay, Roche Diagnostics International, Rotkreuz, Switzerland) and to measure [HCY]_p_ in horses with left-sided valvular heart disease. Our hypothesis was that [HCY] will be stable and quantifiable across sample preparation and storage conditions, making this assay practical for use in a clinical setting. Additionally, considering our pilot data, we hypothesized that there will be a positive association between left-sided valvular regurgitation severity, left-sided cardiac chamber enlargement and [HCY]_p_.

## 2. Materials and Methods

### 2.1. Animals and Blood Samples

Blood samples used in this study were submitted and stored in the respective biobanks of the University of Zurich (UZH), Switzerland and Ghent University (UG), Belgium. According to the regulations of both institutions, no additional ethical approval was required for the use of these samples. All examinations were performed according to institutional ethical standards at UZH and UG. Owners’ consent was obtained for the storage and future anonymized use of blood samples and clinical data.

Healthy horses (used for sample handling studies): A convenience sample of horses that were considered healthy, with no evidence of underlying disease based on history, physical examination including thorough cardiothoracic auscultation, transthoracic echocardiography, resting electrocardiography (ECG) analysis, complete blood count and plasma biochemistry analysis was obtained. All healthy horses were Warmbloods.

Horses with left-sided valvular heart disease: Horses were chosen for inclusion in this study from biobank databases at the UZH and UG. The inclusion criteria were the following: (1) Warmblood breed, (2) complete clinical examination with presence of a left-sided systolic or diastolic heart murmur grade 2/6 or louder, indicating probable left-sided valvular heart disease, and (3) documented history, physical examination including thorough cardiothoracic auscultation, complete transthoracic echocardiography, and resting ECG analysis. Horses with primary right-sided heart disease and congenital defects were excluded. Blood had been sampled as part of a routine clinical workup for cardiac disease and remaining blood was then biobanked. Blood samples for all horses had been collected in lithium-heparin (LH) tubes, spun down within 30 min of collection and plasma separated and stored at −20 °C (UG) or −80 °C (UZH). The UG samples were shipped frozen to UZH and then stored at −80 °C until analysis. Samples were stored for a maximum of two years before analysis.

All samples were analyzed in batches by an automated enzyme assay (Roche Cobas HCYS Homocysteine Enzymatic Assay, Roche Diagnostics International, Rotkreuz, Switzerland) for [HCY]_p_**,** performed according to the manufacturer’s instructions. The measurement range was 3–50 µmol/L; however, the analyzer did report values below 3 µmol/L. When this occurred, the absolute reported value was used. The assay was calibrated at the start of each reagent lot and every 7 days thereafter and reagents were discarded if not used after 4 weeks. Plasma creatinine concentrations ([Creat]_p_) were measured using the compensated Jaffe method run on an established analyzer (Roche Cobas INTEGRA 800 analyzer, Roche Diagnostics International, Rotkreuz, Switzerland).

For the second part of the study, sample size calculations were performed using a G*Power version 3.1.9.7 (G*Power, University Kiel, Germany) ANOVA sample size calculator, to detect a difference in [HCY]_p_ between four groups (horses with trivial, mild, moderate and severe regurgitation); with a medium effect size of 0.25 (derived from unpublished pilot data), alpha error of 0.05 and power of 80%, a total of 180 horses (45 per group) would be required.

### 2.2. Short-Term Stability

To investigate short-term stability of [HCY]_p_ in conditions like those found in clinical practice, blood samples from 2 healthy horses were collected in LH tubes via jugular venipuncture as part of their general workup. One aliquot of each horse was centrifuged immediately to obtain plasma. One aliquot of anticoagulated whole blood (no centrifugation) was stored at room temperature. Aliquots of plasma were stored at room temperature and at 4 °C, respectively. Homocysteine concentrations in all aliquots were measured at the 0 h timepoint (shortly after blood collection), and following storage for 1 h, 2 h, 4 h, 8 h, and 24 h. Anticoagulated whole blood samples were centrifuged following the storage time and plasma was used for the HCY assay. All [HCY]_p_ measurements were performed in triplicate.

### 2.3. Long-Term Stability

To investigate long-term stability of [HCY]_p_, LH plasma samples of 8 horses from the UZH cardiology biobank (stored at −80 °C) were analyzed and [HCY]_p_ was compared to results measured from the same samples, 2 years previously. The first [HCY]_p_ had been measured in single runs, whereas repeated [HCY]_p_ were performed in triplicate.

### 2.4. Influence of Anticoagulants

To investigate the influence of different anticoagulants on [HCY], stored samples from the UZH cardiology biobank were selected, originating from 19 horses, for which blood had been collected in four different blood tubes (VACUETTE^®^, Greiner Bio-One, St. Gallen, Switzerland) (no additive for serum, LH, sodium citrate, EDTA). Plasma or serum [HCY] were analyzed in duplicate.

### 2.5. Influence of Repeated Freeze–Thaw Cycles

To investigate the influence of repeated freeze–thaw cycles on [HCY]_p_, fresh blood samples of 6 healthy horses were collected in LH tubes as part of their general work up. The blood samples were centrifuged and plasma was collected. Following centrifugation, the [HCY]_p_ for timepoint 0 was measured. The plasma was then placed in −80 °C for at least 6 h and [HCY]_p_ was remeasured after rethawing. Samples were then refrozen and the process repeated. All [HCY]_p_ measurements were performed in duplicate, with 5 freeze–thaw repeats.

### 2.6. Clinical and Echocardiographic Examinations

Clinical examination findings were extracted from the medical databases of the UZH and the UG. Cardiac murmurs had been classified according to point of maximal intensity (PMI), timing within the cardiac cycle, and intensity on a scale of 0–6/6 [37].

Transthoracic echocardiographic examinations, including 2-dimensional (2D), motion mode (M-mode) and color flow Doppler had been performed with GE (GE Healthcare, Freiburg, Germany) e95 echocardiograph using an M5Sc (UZH) probe or a GE Vivid 7 echocardiograph using a 3S (UG) probe by multiple trained veterinarians at each institution and according to a standardized protocol. Raw-data recordings were extracted from the GE EchoPac databases of the UZH and the UG and re-measured offline for the purpose of this study by two experienced observers (KJM and EWL). Cardiac structures, valvular competence, chamber dimensions, left atrial (LA) contractile function, and left ventricular (LV) systolic function were assessed using right and left parasternal long-axis and short-axis views [22,38,39,40]. Three representative, cardiac cycles were measured and averaged for each variable.

The 2D echocardiographic variables used to assess LA size and function included the following: LA diameter at end-systole (LAD_rlx-max_), LA area at end-systole (LAA_rlx-max_), at the onset of the electrocardiographic P wave (LAA_rlx-a_), and at the time of closure of the mitral valve (LAA_rlx-min_); in a right parasternal long-axis view; LA area at end-systole (LAA_rsx-max_) in a right parasternal short-axis view; and LA diameter at end-systole (LAD_llx-max_) measured in a left parasternal long-axis view. Active LA fractional area change was calculated as active LA FAC = (LAA_rlx-a_ − LAA_rlx-min_)/LAA_rlx-a_ × 100) [39].

Variables used to assess LV size and function included the following: internal diameter of the LV at end-diastole (LVID_d_) and peak-systole (LVID_s_), diameter of the interventricular septum at end-diastole (IVS_d_) and peak-systole (IVS_s_), diameter of the LV free wall at end-diastole (LVFW_d_) and peak-systole (LVFW_s_), relative left ventricular wall thickness at end-diastole (RWT_d_ = (IVS_d_ + LVFW_d_)/LVID_d_), mean left ventricular wall thickness at end-diastole (MWT_d_ = (IVS_d_ + LVFW_d_)/2), and LV fractional shortening (LV FS = (LVID_d_ − LVID_s_)/LVID_d_ × 100) in a right-parasternal short-axis M-mode recording at the chordal level; and, LV internal volume at end-diastole (LVIV_d_) and peak-systole (LVIV_s_), LV ejection fraction (LV EF = (LVIV_d_ − LVIV_s_)/LVIV_d_ × 100), stroke volume (SV = LVIV_d_ − LVIV_s_) and cardiac output (CO = SV × HR/1000) in a right parasternal long-axis view using single-plane Simpson’s model of disks [40].

Measurements of the great vessels included the following: aortic sinus diameter at end-diastole (AoD_rlx-ed_) in a right parasternal long-axis view of the left ventricular outflow tract, aortic area at end-systole (AoA_rsx-es_) in a right parasternal short-axis view at the level of the aortic valve, and sinus diameter of the pulmonary artery at end-diastole (PAD_rlx-ed_) in a right parasternal long-axis view of the right ventricular outflow tract [41].

Measurements of great vessels and chamber dimensions were corrected for differences in body mass using previously defined equations for allometric scaling of echocardiographic variables in horses [22,39,40]. Specifically, the measurements were normalized to a body weight (BWT) of 500 kg using the following equations: diameter [500] = measured diameter/BWT^1/3^ × 500^1/3^; area [500] = measured area/BWT^2/3^ × 500^2/3^; and volume [500] = calculated volume/BWT × 500.

The severity of valvular insufficiency seen on color flow Doppler imaging was categorized based on subjective assessment of the size of the regurgitant jet size within the respective receiving chamber (i.e., left atrium or left ventricular outflow tract) and timing within the cardiac cycle (systole or diastole). The categories were defined as follows: trivial (regurgitant jet only visible in some frames, not all of systole or diastole), mild (regurgitant jet ≤ 1/4 of receiving chamber, duration of systole or diastole), moderate (regurgitant jet > 1/4 and <3/4 of receiving chamber), or severe (regurgitant jet ≥ 3/4 of receiving chamber) [42]. Horses with multiple valve regurgitations were grouped according to the highest severity grading recorded. A cardiac chamber was considered enlarged if one or more of its dimensional measurements were outside the upper end of the respective reference interval when normalized for 500 kg [39,40].

### 2.7. Statistical Analysis

Commercially available software was used for all statistical and graphical analysis (Microsoft Excel 365 (Microsoft, Redmond, WA, USA), GraphPad Prism v10.4.0 (Graphpad Software Inc., Boston, MA, USA), R v4.4.2, R Studio v2024.12.0 + 467 (R and RStudio, Posit Software, Boston, MA, USA)). Descriptive statistics were calculated. Data were visually inspected for normality using QQ plots and histograms. Normally distributed data were expressed as a mean ± standard deviation (SD), while non-normally distributed data were expressed as a median and range. Two-way repeated measures analysis of variance (ANOVA) with Tukey’s multiple comparison post hoc tests were performed to assess short-term stability, a one-way repeated measures ANOVA was performed to evaluate freeze–thaw stability of [HCY]_p_, Wilcoxon matched-pairs signed rank test was used to compare long-term stability, and a Friedman test with Dunn’s multiple comparison test was used to compare [HCY] measured using different anticoagulants.

Associations between echocardiographic variables, [Creat]_p_, age and [HCY]_p_ were first investigated with a Pearson’s correlation coefficient (normally distributed variables) or Spearman’s correlation coefficient (non-normally distributed variables), and linear regression goodness-of-fit was calculated for each correlation in Graphpad Prism.

Association between [HCY]_p_, age, sex, regurgitation severity and echocardiographic variables were then assessed using linear models (R packages car-companion to applied regression and emmeans-estimated marginal means). Homocysteine data were not normally distributed, therefore log transformation was performed and data were subsequently found to be normally distributed based on visual inspection of QQ plots and histograms. Approximately half the echocardiographic data were normally distributed; however, all echocardiographic variables were expressed as median (range) so as to more easily compare them with the appropriate reference intervals. Models were assessed for collinearity using the variance inflation factor and normality of the residuals were assessed using histograms and dot plots. Models with low collinearity (<5) and normal residuals were considered representative. Derived/calculated variables (e.g., RWT_d_, MWT_d_, FS) were removed from the final LV models due to collinearity with measured LV variables. Details of the models are found in Supplementary Table S2.

The level of significance was set at *p* < 0.05 for all statistical tests.

## 3. Results

### 3.1. Analyte Stability and Influence of Anticoagulants

Homocysteine concentration varied with storage management. At 8 h, [HCY]_p_ were higher when samples were stored at room temperature compared to 4 °C, regardless of whether samples were stored as whole blood or plasma (mean difference from baseline, +0.2 µmol/L for both, both *p*-values = 0.0233). At 24 h [HCY]_p_ samples stored at room temperature as whole blood were statistically significantly higher compared to plasma stored both at 4 °C and room temperature (mean difference from baseline, +0.3 µmol/L, both *p*-values = 0.0024).

**Figure 1:**
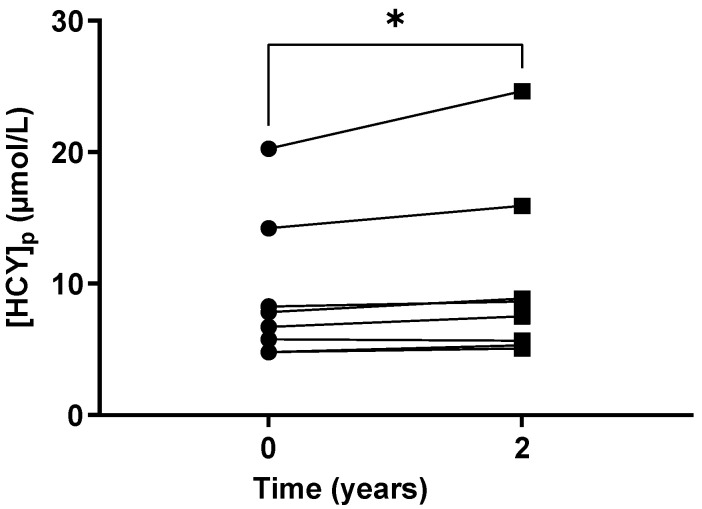
Long-term stability of plasma homocysteine concentrations ([HCY]_p_). Timepoint 0 measured at the time of blood collection and timepoint 2 measured 2 years later after being stored at −80 °C. The median difference between timepoint 0 and 2 was 0.68 µmol/L (range: −0.1 to 4.4 µmol/L, * *p* = 0.0156).

The [HCY]_p_ in LH-anticoagulated plasma samples increased after being stored in −80 °C for two years (Figure 1).

**Figure 2:**
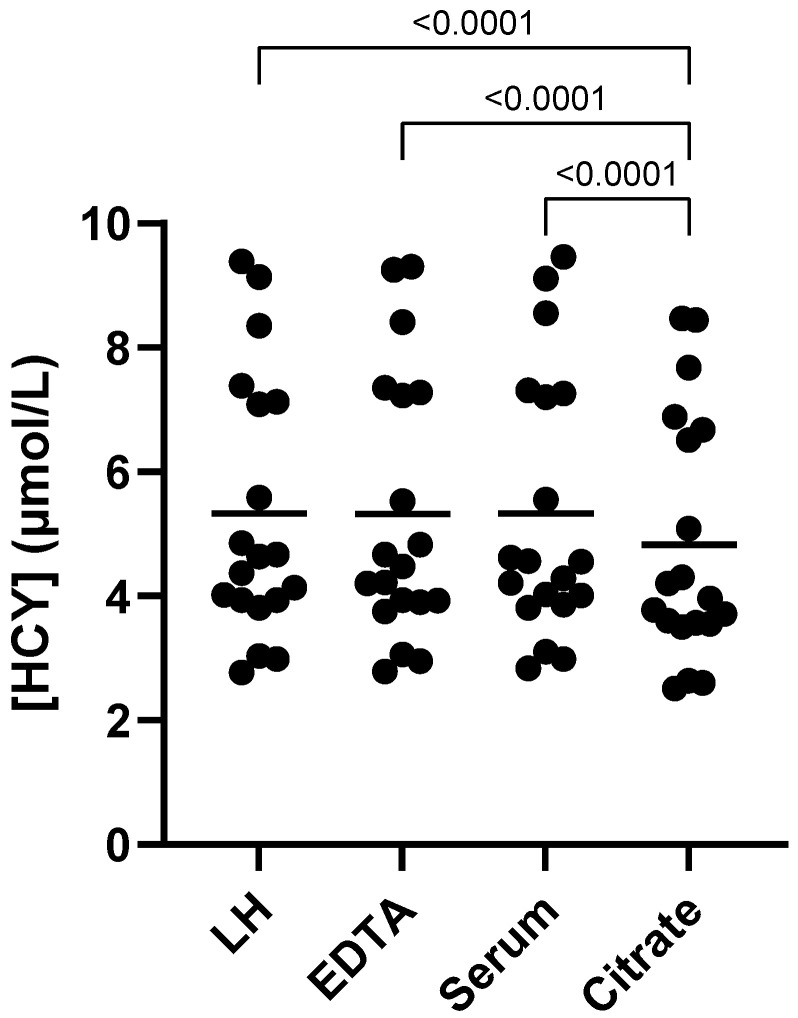
Influence of different anticoagulants on homocysteine concentration [HCY] in 19 horses. The [HCY] in sodium citrate samples was lower compared to all other samples. No significant differences were detected in [HCY] between LH plasma, EDTA plasma and serum.

Comparing the different anticoagulants, no significant differences were detected for [HCY]_p_ in LH and EDTA-anticoagulated plasma and for serum [HCY]. However, [HCY]_p_ in sodium citrate-anticoagulated plasma was significantly lower compared to the other three groups (Figure 2).

**Figure 3:**
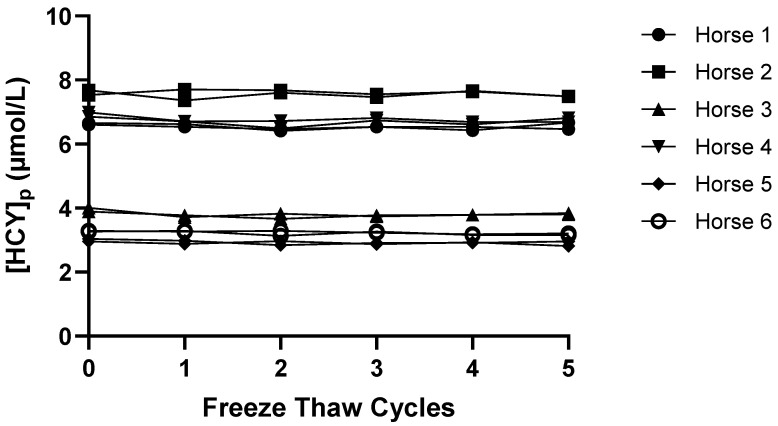
Plasma homocysteine concentrations ([HCY]_p_) over 5 freeze–thaw cycles. No statistical difference in [HCY]_p_ was noted between any of the time points (F-test, *p* = 0.059).

The [HCY]_p_ in LH-anticoagulated plasma concentrations remained stable over the duration of five freeze–thaw cycles (Figure 3).

### 3.2. Study Sample—Left-Sided Valvular Heart Disease

The samples originated from horses that had been presented to UZH and UG for cardiac evaluation between October 2016 and July 2018. Data from a total of 169 Warmblood horses were evaluated for association between left-sided valvular regurgitation, echocardiographic indices of left atrial and left ventricular size and function, and [HCY]_p_. The study sample included 139 horses from UG (nine with trivial and 130 with mild or greater left-sided valvular regurgitation) and 30 horses from UZH (eight with trivial and 22 with mild or greater left-sided valvular regurgitation). Fifty horses were female and 119 horses were male (13 stallions, 106 geldings). The median age of the horses was 8 years (range 2–27 years), their weight was 568 ± 52 kg (mean ± SD).

### 3.3. Homocysteine Plasma Concentrations and Left-Sided Valvular Heart Disease

**Figure 4:**
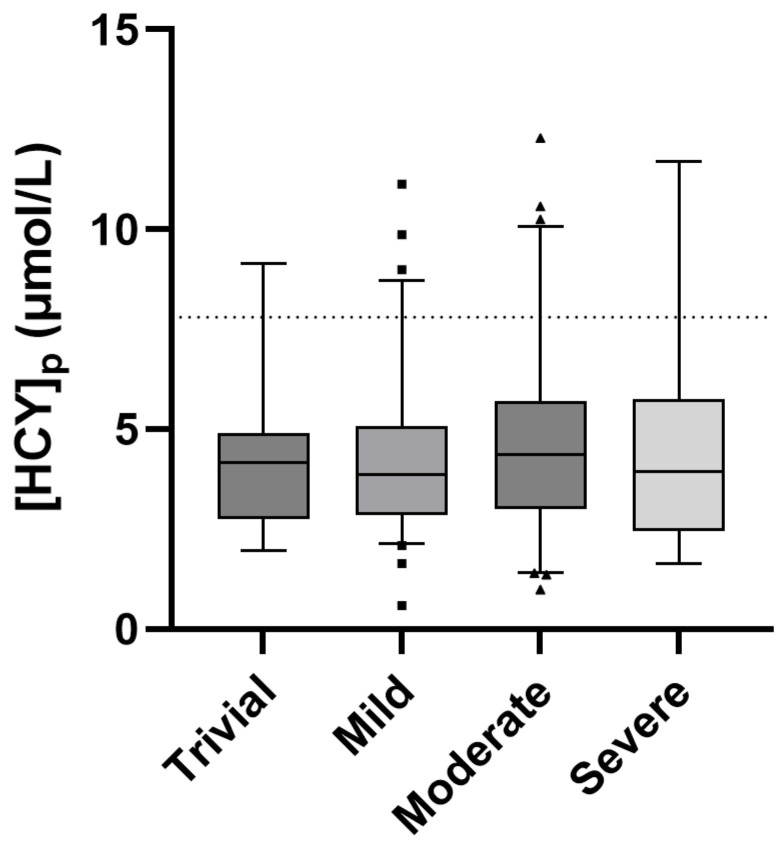
Plasma homocysteine concentrations ([HCY]_p_) grouped by valvular regurgitation severity assessed by color Doppler receiving chamber analysis. Horses with trivial (*n* = 17), mild (*n* = 70), moderate (*n* = 65) or severe (*n* = 17) left-sided regurgitation (mitral, aortic or both) were grouped together. Box-and-whisker plot represents median, quartiles and 5th/95th percentiles. Outliers (<5%/>95%) are displayed as individual points. The dotted line represents the upper reference interval for [HCY]_p_ in healthy horses [28]. No significant differences in [HCY]_p_ were detected between groups (Supplementary Table S2).

The median [HCY]_p_ concentration for the 169 horses was 4.1 μmol/L (0.6–12.3 μmol/L). One hundred and fifty-two horses were classified using color Doppler as having mild or greater left-sided regurgitation, 91 horses with mitral regurgitation, 30 horses with aortic regurgitation and 31 horses with both. The remaining 17 horses were diagnosed with trivial left-sided regurgitation. The [HCY]_p_ for these four groups are visualized in Figure 4. Most horses were in sinus rhythm, with 17 horses in sustained atrial fibrillation and two horses with sinus tachycardia secondary to their underlying cardiac disease.

The summary of the echocardiographic findings from all horses can be found in Table 1. There were 47 horses with only an enlarged LA and there were six horses with only an enlarged LV, 14 horses had both LA and LV enlargement.

**Figure 5:**
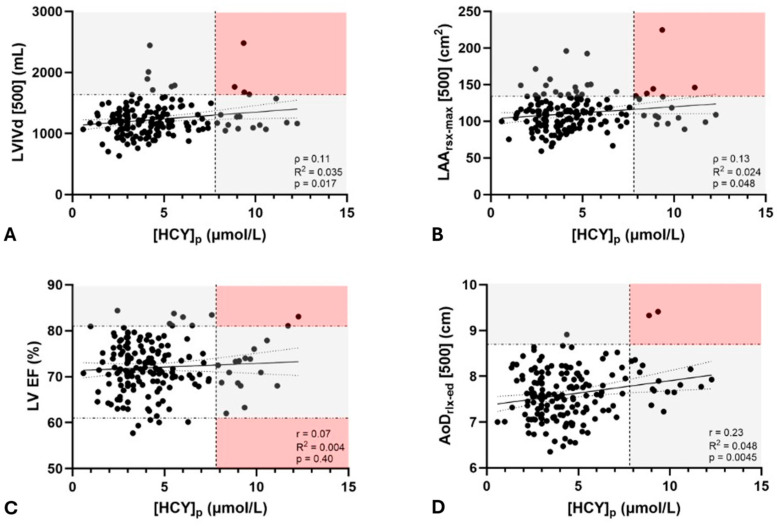
(**A**–**D**) Representative echocardiography indices of (**A**) left ventricular volume (LVIV_d_ [500]), (**B**) maximal left atrial area in short axis (LAA_rsx-max_ [500]), (**C**) left ventricular ejection fraction (% LV EF) and (**D**) aortic sinus diameter (AoD_rlx-ed_ [500]) and the association with plasma homocysteine concentrations ([HCY]_p_). The horizontal dashed line represents the relevant limits of the reference interval in Warmblood horses [39,40], and the vertical dashed line represents the upper reference limit for [HCY]_p_ from healthy Warmblood horses [28]. The red shaded areas indicate horses that had both an echocardiographic variable measuring outside of the reference interval and elevated [HCY]_p_. The gray shaded areas include individuals with an increased echocardiographic variable but normal [HCY]_p_ or a normal echocardiographic variable and elevated [HCY]_p_. The solid line represents regression line, and the dotted lines either side represent the 95% confidence intervals of the regression line. Abbreviations: % LV EF, % left ventricular ejection fraction; AoD_rlx-ed_ [500], aortic sinus diameter in a right parasternal long-axis view of the left ventricular outflow tract at end-diastole, normalized to 500 kg; [HCY]_p_, plasma homocysteine concentration; LAA_rsx-max_ [500], left atrial maximum area in a right parasternal short-axis view, normalized to 500 kg; LVIV_d_ [500], left ventricular internal volume at end-diastole, normalized to 500 kg; r, Pearson’s correlation coefficient; ρ, Spearman’s correlation coefficient; and R^2^, linear regression coefficient of determination.

**Figure 6:**
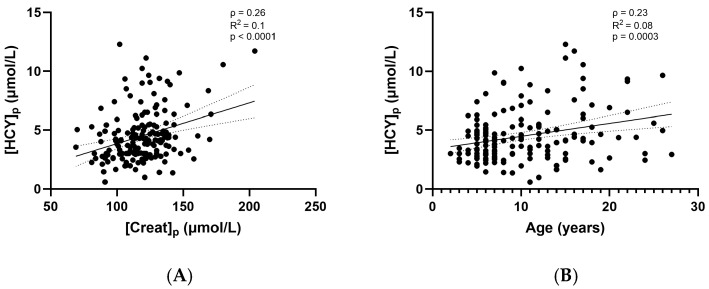
(**A**), Correlation between plasma homocysteine concentration ([HCY]_p_) and plasma creatinine concentration ([Crea]_p_) (*n* = 169). (**B**), Correlation between [HCY]_p_ and age (*n* = 169). ρ, Spearman’s correlation coefficient, R^2^, linear regression coefficient of determination. The solid line represents regression line, and the dotted lines either side represent the 95% confidence intervals of the regression line.

The correlation analyses showed very weak significant correlations between [HCY]_p_ and LVIDd [500], LVIDs, LVIVd [500], LAA_rsx-max_ [500], PAD_rlx-ed_ [500], AoD_rlx-ed_ [500] and AoA_rsx-es_ [500]. The correlation analyses for [Creat]_p_, age and [HCY]_p_ were weak but significant. Correlation and linear regression coefficients for each echocardiographic variable and [HCY]_p_ can be found in Supplementary Table S1. Representative graphs of LA size, LV size, LV function and aortic dimensions correlated with [HCY]_p_ are seen in Figure 5, while graphs of [Creat]_p_ and age correlated with [HCY]_p_ are seen in Figure 6.

Eight linear models evaluating the association of [HCY]_p_ with age, signalment (sex, weight), echocardiographic indices of left atrial and left ventricular size and function and great vessels measurements, regurgitation severity, and [Creat]_p_ were evaluated (see Supplementary Table S2 for specific details). The models investigating left atrial and left ventricular size and function showed no significant association of [HCY]_p_ with any of the variables. The model investigating sex, weight, age, regurgitation severity and [Creat]_p_ showed significant positive associations between age, [Creat]_p_ and [HCY]_p_ (Figure 6). No interaction or collinearity between age and [Creat]_p_ was observed.

Post hoc, the actual effect size and study power was calculated with the collected data (trivial, mild, moderate and severe regurgitation groups). The effect size was 0.08 (small) with power of 13%. Given the measured effect size, a study sample of 1708 horses (427/group) would have been required to detect a difference between these groups.

## 4. Discussion

This study describes sample handling, processing and storage conditions relevant for measuring [HCY] in equine blood samples with an automated HCY enzyme-cycling assay. Furthermore, the study investigated the association between echocardiographic variables of left heart size and function and [HCY]_p_ in horses with left-sided valvular regurgitation. While equine [HCY]_p_ appears to have good short- and long-term stability and the HCY assay is not affected by the use of common anticoagulants, [HCY] is of limited diagnostic utility as a biomarker in horses with left-sided valvular heart disease.

### 4.1. Analyte Stability and Influence of Anticoagulants

Storage of equine whole blood at room temperature for up to 4 h did not affect [HCY]_p_, which allows feasible use of the established HCY assay in a clinical setting. Storing whole blood samples at room temperatures for longer time periods however, affected [HCY]_p_ significantly. This is consistent with the results of a study in humans, where [HCY]_p_ samples were stable for up to 4 h, whereas [HCY]_p_ increased by 60% over 24 h when stored at room temperature [4,43]. Delayed centrifugation has been identified as a cause for increased [HCY]_p_, thought to be due to the release of HCY from erythrocytes [44]. For blood samples submitted for HCY determination, spinning and separation of serum or plasma as soon as possible is recommended.

At low [HCY]_p_, samples appear to remain stable for at least two years when stored at −80 °C. This result is consistent with previous studies in people where [HCY]_p_ remained stable for several years regardless of storage temperature (−20 or −80 °C) [45]. Samples with high [HCY]_p_ (2–3 times above recommended reference range [28]) may be less stable when stored at −80 °C for two years (Figure 1). The limited number of samples with high [HCY]_p_ in this sample should be considered when interpreting these results.

Homocysteine concentrations were significantly lower when samples were processed with sodium citrate anticoagulant (Figure 2). Previous studies have also identified [HCY]_p_ samples stored with sodium citrate differing from samples stored in EDTA [4,46]. There was no difference in [HCY] between serum, EDTA, and LH samples (Figure 2). This contrasts findings of a previous study, identifying higher [HCY] in serum compared to plasma [4,43]. This contrast might be explained by the samples in this current study being centrifuged in a timely manner. Delayed centrifugation has been demonstrated to lead to increased [HCY]_p_ due to release from erythrocytes [44]. However, subtle differences could also be undetected due to small sample size. In dogs, comparison of [HCY] in serum, EDTA, and LH samples identified serum as the preferred sample for HCY testing [29]. This has also been concluded in a publication reviewing methods for HCY testing in humans [4].

Once LH-anticoagulated equine plasma samples are frozen, [HCY]_p_ were shown to remain stable within 5 freeze–thaw cycles (Figure 3). Stability of [HCY]_p_ over several freeze–thaw cycles has previously been shown in human plasma [45,47].

### 4.2. Associations Between Plasma Homocysteine Concentration, Age and Plasma Creatinine Concentration

A positive association between age, [Creat]_p_ and [HCY]_p_ was identified in this study. In humans, [HCY]_p_ were associated with increased [Creat]_p_ and age among other factors [4]. Homocysteine is physiologically excreted via the kidneys [48] and even mild reduction in glomerular filtration rate may contribute to increased [HCY]_p_ before overt azotemia develops. Therefore, increased [HCY]_p_ in some horses may be secondary to concurrent subclinical renal injury and decreased glomerular filtration. Due to the retrospective nature of this part of the study, concurrent renal disease in older horses could not be excluded, but no clear interaction or collinearity between age and [Creat]_p_ was found. In addition to decreased renal function, authors of previous studies have speculated that age-related increase of [HCY]_p_ may be associated with decreased liver function, as well as digestive and absorption dysfunctions, leading to vitamin B_12_ and folate deficiencies, both leading to decreased HCY metabolism and subsequent accumulation [49]. Hyperhomocysteinemia has been demonstrated to occur more often in elderly humans due to decreased HCY metabolism [13,49]. In a previous study in horses, [Creat]_p_ and [HCY]_p_ were associated independent of age [28], which is slightly discordant with the current findings, possibly due to the larger sample size of this current study. In people, there is increasing evidence that HHCY is not only a marker of age and disease but also accelerates aging and contributes to cardiovascular disease [8,13]. Future research is needed to investigate [HCY]_p_ in horses with confirmed renal pathology as well as the influence of vitamin B_12_ and folate status.

### 4.3. Associations with Homocysteine and Left-Sided Valvular Heart Disease

Despite this study containing a fairly large sample size of horses with left-sided valvular regurgitation and cardiac enlargement, there were no associations found between [HCY]_p_ and any echocardiographic variables of cardiac enlargement or abnormal function in horses once the echocardiographic variables were combined in the various models. This contrasts with human medicine, where there is evidence of associations between [HCY]_p_ and left atrial and left ventricular enlargement as well as left ventricular dysfunction, detected on echocardiography [14,15,20,50,51,52,53]. While this current study lacked large numbers of horses with severe regurgitation in cardiac failure, many horses had moderate and severe valvular regurgitation with a degree of cardiac chamber enlargement. This highlights key differences in the pathophysiology of left-sided valvular heart disease between humans and horses. In humans, factors such as diet, exercise, history of tobacco usage and alcohol intake are all related to HHCY and cardiovascular disease. In horses, lifestyle factors, atherosclerotic vascular disease, and underlying cardiomyopathies do not play a relevant role, and valvular regurgitation is primarily associated with age-related degeneration and in most cases does not progress to heart failure. The findings of this study support those of Mitchell et al. 2018, where [HCY]_p_ was not associated with the development or recurrence of equine atrial fibrillation, unlike that is seen in people [20,28]. This evidence suggests that [HCY]_p_ is not a suitable biomarker for detection of left-sided valvular heart disease in horses.

### 4.4. Limitations

Some limitations must be considered when interpreting the results of this study. A convenience sample was used for analyte stability and influence of anticoagulants, and no sample size calculation was performed for this part of the study. Therefore, small differences could have been missed. Echocardiographic images were obtained by different operators at two institutions. To reduce variability of echocardiographic measurements, all images were measured specifically for this study by one of two experienced operators (KJM and EWL).

The severity of valvular regurgitation was categorized based on subjective assessment of the size of the regurgitant jet size within the respective receiving chamber visualized with color flow Doppler imaging, a method that has been used for many years. Although not fully validated, scoring systems have recently been proposed for both AR and MR severity in horses, allowing a more standardized evaluation and taking into consideration any chamber enlargement in addition to the regurgitant jet size. The data for this current study was already analyzed before these were developed and therefore the proposed scoring systems were not implemented here.

The sample size calculation performed with the pilot data suggested that the total number of horses needed to be 180, with 45 horses in each group. Due to the retrospective use of biobanked samples, we only reached a total of 169 horses, which were not equally distributed among the severity groups. Furthermore, the post hoc power and effect size calculation shows that the actual effect size was much smaller than the pilot data had suggested, resulting in an underpowered study. Given the measured effect size, a study sample of 1708 horses (427/group) would have been required, which is not a feasible number in most equine cardiac biomarker studies. Considering the limitations addressed above, a very small association between [HCY]_p_ and left-sided valvular heart disease cannot be completely ruled out. However, there is likely limited biological relevance with this small magnitude of difference.

## 5. Conclusions

Plasma [HCY] remains stable for up to 4 h, independent of storage management, allowing the use of this assay in a clinical setting. If samples cannot be immediately spun and plasma separated, they should be cooled at 4 °C and then separated at the earliest possibility. Sodium citrate will result in lower [HCY]_p_ when using this assay and therefore should be avoided. Long-term (>2 years) storage of samples with higher [HCY]_p_ is not recommended as [HCY]_p_ increased over time, even at −80 °C. This is unlikely to affect the clinical application but should be considered in a research setting.

Plasma [HCY]_p_ does not appear increased in horses with left-sided valvular regurgitation but is positively associated with [Creat]_p_ and age. The usefulness of [HCY]_p_ as a biomarker for structural or functional cardiac changes in horses is therefore limited. As horses appear to have alternative pathophysiological mechanisms of developing left-sided valvular heart disease, direct comparisons with human HCY-mediated pathophysiological mechanisms should be avoided.

## Figures and Tables

**Table 1:** Summarized echocardiographic variables of all horses included in the linear models, with data presented as median (range) and compared to the relevant reported reference intervals.

Echocardiographic Variables		Median (Range) *n* = 169	Reported Reference Intervals [39,40]
**LV Size and Function**			
IVS_d_	cm	3.0 (2.2–4.0)	2.4–3.5
IVS_s_	cm	4.3 (2.4–5.4)	3.6–5.4
LVID_d_	cm	12.0 (9.9–16.6)	9.7–13.4
LVID_d_ [500]	cm	11.6 (9.4–17.4)	9.2–12.9
LVID_s_	cm	7.2 (4.5–10.8)	4.7–9.1
LVFW_d_	cm	2.6 (1.7–4.0)	1.8–3.4
LVFW_s_	cm	4.4 (3.1–6.2)	3.6–5.8
% FS	%	40 (23–59)	27–54
MWT_d_	cm	2.8 (1.8–3.7)	2.3–3.2
RWT_d_		0.46 (0.30–0.67)	0.36–0.62
LVIV_d_	mL	1324 (663–3257)	1057–1893
LVIV_d_ [500]	mL	1187 (633–2482)	954–1638
LVIV_s_	mL	366 (156–945)	241–582
% EF	%	72 (58–84)	61–81
SV	mL	974 (465–2312)	774–1356
HR	bpm	42 (28–94)	27–53
CO	L/min	42.5 (15.7–102.5)	25.1–58.4
**LA Size and Function**			
LAD_rlx-max_ [500]	cm	12.3 (9.8–16.5)	10.5–13.2
LAD_llx-max_ [500]	cm	13.2 (11–20.1)	11.8–14.0
LAA_rlx-max_ [500]	cm^2^	98.5 (66.3–187.6)	82.3–103.2
LAA_rsx-max_ [500]	cm^2^	108.9 (59.4–224.8)	83.5–134.1
Active LA FAC	%	22 (−12–40)	6–33
**Great Vessels**			
PAD_rlx-ed_ [500]	cm	5.9 (4.3–9.5)	5.6–7.4
AoD_rlx-ed_ [500]	cm	7.6 (5.2–9.4)	6.5–8.7
AoA_rsx-es_ [500]	cm^2^	45.6 (30.2–67.2)	33.5–56.2

Abbreviations: %EF, % ejection fraction; %FS, % fractional shortening; active LA FAC, active left atrial fractional area change; AoA_rsx-es_ [500], aortic area in a right parasternal short-axis view at the level of the aortic valve at beginning of diastole, normalized to 500 kg; AoD_rlx-ed_ [500], aortic diameter in a right parasternal long-axis view of the left ventricular outflow tract at end-diastole, normalized to 500 kg; CO, cardiac output; IVS_d_ and IVS_s_, diameter of the interventricular septum at end-diastole and peak-systole; LA, left atria; LAA_rlx-max_ [500], maximum left atrial area in a right parasternal long-axis view at end-systole, normalized to 500 kg; LAA_rsx-max_ [500] left atrial area in right parasternal short-axis at beginning of diastole, normalized to 500 kg; LAD_rlx-max_ [500] and LAD_llx-max_ [500], maximum left atrial diameter measured at end-systole from the right and the left, normalized to 500 kg; LV, left ventricle; LVFW_d_ and LVFW_s_, diameter of the left ventricular free wall at end-diastole and peak-systole; LVID_d_, and LVID_s_, LVID [500], internal diameter of the LV at end-diastole and peak-systole and normalized to 500 kg; LVIV_d_, LVIV_s_, LVIV_d_ [500], internal volume of the LV at end-diastole, peak-systole and normalized to 500 kg; LVIV_s_, left ventricular internal volume at peak-systole; MWT_d_, mean left ventricular wall thickness at end-diastole; PAD_rlx-ed_ [500], diameter of the pulmonary artery at end-diastole normalized to 500 kg; RWT_d_, relative left ventricular wall thickness at end-diastole; SV, stroke volume.

## Data Availability

Data is available on request to the corresponding author.

## Supplementary Materials

The following supporting information can be downloaded at https://www.mdpi.com/article/10.3390/ani16152307/s1, Table S1: Correlation and linear regression coefficients when comparing plasma homocysteine concentrations ([HCY]_p_) to each of the echocardiographic variables; Table S2: Details of linear models investigating associations between age, sex, weight, creatinine, regurgitation severity and echocardiographic variables and plasma homocysteine concentrations.

## Abbreviations

The following abbreviations are used in this manuscript:

AF	Atrial fibrillation
ANOVA	Analysis of variance
AoD_rlx-ed_	Aortic sinus diameter in a right parasternal long-axis view at end-diastole
AoA_rsx-es_	Aortic area in a right parasternal short-axis view at end-diastole
BWT	Body weight
CO	Cardiac output
[Creat]_p_	Plasma creatinine concentrations
ECG	Electrocardiogram
EDTA	Ethylenediaminetetraacetic acid
HCY	Homocysteine
[HCY]_p_	Plasma homocysteine concentrations
HHCY	Hyperhomocysteinemia
IVS_d_	Diameter of the interventricular septum at end-diastole
IVS_s_	Diameter of the interventricular septum at peak-systole
LA	Left atrium
LAD_rlx-max_	LA diameter in a right parasternal long-axis view at end-systole
LAA_rlx-max_	LA area in a right parasternal long-axis view at end-systole
LAA_rlx-a_	LA area in a right parasternal long-axis view at the onset of the P wave
LAA_rlx-min_	LA area in a right parasternal long-axis view at the time of closure of the mitral valve
LAA_rsx-max_	LA area in a right parasternal short-axis view at end-systole
LAD_llx-max_	LA diameter in a left parasternal long-axis view at end-systole
LA FAC	Active LA fraction area change
LH	Lithium heparin
LV	Left ventricle
LV EF	LV ejection fraction
LV FS	LV fractional shortening
LVFW_d_	Diameter of the LV free wall at end-diastole
LVFW_s_	Diameter of the LV free wall at peak-systole
LVID_d_	Internal diameter of the LV at end-diastole
LVID_s_	Internal diameter of the LV at peak-systole
LVIV_d_	LV internal volume at end-diastole
LVIV_s_	LV internal volume at peak-systole
M-mode	Motion mode
MWT_d_	Mean LV wall thickness at end-diastole
PAD_rlx-ed_	Sinus diameter of the pulmonary artery in a right parasternal long-axis view at end-diastole
PMI	Point of maximal intensity
RWT_d_	Relative left ventricular wall thickness at end-diastole
SD	Standard deviation
SV	Stroke volume
UG	Ghent University
UZH	University of Zurich
2D	2-dimensional
