# Supplementary material for: Plasma Homocysteine Concentrations in Horses with Left-Sided Valvular Heart Disease
Source: Animals (Basel). 2026 Jul 26;16(15):2307. doi: 10.3390/ani16152307 (PMC13463591; doi:10.3390/ani16152307)
Supplement: Supplementary file 1 [file animals-16-02307-s001.zip › Supplementary Table S1 Correlation data 2026-02-22.pdf]

Supplementary Table S1.

Correlation and linear regression coefficients when comparing plasma homocysteine concentrations ($[HCY]_p$) to each of the echocardiographic variables.

	Pearson correlation coefficient (r)	Spearman correlation coefficient (ρ)	Linear regression (R^2)	P value
**LV size and function**				
IVS_d	0.048		0.0023	0.54
IVS_s		-0.11	0.0080	0.25
$LVID_d$ [500]		0.066	0.024	**0.049**
$LVID_s$	0.18		0.032	**0.020**
$LVFW_d$	0.049		0.0024	0.53
$LVFW_s$	0.023		0.00053	0.77
% FS	-0.14		0.021	0.062
MWT_d	0.052		0.0027	0.51
RWT_d		-0.045	0.0018	0.59
LVIV_d [500]		0.11	0.035	**0.017**
LVIV_s		0.084	0.0090	0.22
% EF	0.065		0.0042	0.40
SV		0.15	0.019	0.075
CO		0.019	0.0034	0.45
**LA size and function**				
$LAD_{rlx-max}$ [500]		0.10	0.014	0.13
$LAD_{llx-max}$ [500]		0.13	0.022	0.058
$LAA_{rlx-max}$ [500]		0.037	0.013	0.15
$LAA_{rsx-max}$ [500]		0.13	0.024	**0.048**
Active LA FAC	0.012		0.00015	0.89
**Great Vessels**				
PAD_{rlx-ed} [500]		0.11	0.025	**0.0499**
AoD_{rlx-ed} [500]	0.22		0.048	**0.0045**
AoA_{rsx-es} [500]	0.18		0.031	**0.025**
