# Supplementary material for: Plasma Homocysteine Concentrations in Horses with Left-Sided Valvular Heart Disease
Source: Animals (Basel). 2026 Jul 26;16(15):2307. doi: 10.3390/ani16152307 (PMC13463591; doi:10.3390/ani16152307)
Supplement: Supplementary file 1 [file animals-16-02307-s001.zip › Supplementary Table S2 - Model details 2026-03-01.pdf]

Supplementary Table S2: Details of linear models investigating associations between age, sex, weight, creatinine, regurgitation severity and echocardiographic variables and plasma homocysteine concentrations.

**Model**	**Predictors**	**Estimate**	**Standard Error**	**T**	**P**	**R^2**
Model 1 (signalment, regurgitation severity, weight, creatinine)	Intercept	0.669	0.445	1.504	0.135	0.150
	Regurgitation-Mild	-0.007	0.131	-0.005	0.996	
	Regurgitation-Moderate	0.046	0.135	0.338	0.736	
	Regurgitation-Severe	-0.118	0.168	-0.697	0.487	
	Age	0.018	0.007	2.488	**0.013**	
	Sex- FC	0.035	0.483	0.072	0.943	
	Sex-M	-0.118	0.115	-1.023	0.308	
	Sex- MC	-0.144	0.085	-1.703	0.091	
	Weight	-0.001	0.007	-0.728	0.468	
	Creatinine	0.008	0.002	4.104	**<0.001**	
Model 2 (LA area, LA function)	Intercept	0.673	0.615	1.094	0.276	0.043
	Weight	0.0001	0.0009	0.165	0.869	
	$LAA_{rlx-max}$ [500]	0.0015	0.004	0.350	0.727	
	$LAA_{rsx-max}$ [500]	0.004	0.003	1.349	0.180	
	Active LA FAC	0.003	0.005	0.499	0.618	
Model 3 (LA diameter)	Intercept	0.361	0.693	0.521	0.603	0.021
	Weight	0.0001	0.0008	0.068	0.945	
	$LAD_{rlx-max}$ [500]	0.010	0.049	0.203	0.839	
	$LAD_{llx-max}$ [500]	0.066	0.055	1.207	0.229	
Model 4 (LV diameters)	Intercept	0.475	0.633	0.750	0.454	0.040

	IVS_d	0.126	0.127	0.986	0.326	
	IVS_s	-0.051	0.063	-0.809	0.420	
	$LVID_s$ [500]	0.025	0.052	0.486	0.628	
	$LVID_s$	0.041	0.052	0.789	0.431	
	$LVFW_d$	0.057	0.108	0.528	0.598	
Model 5 (LV volumes, function)	Intercept	0.931	0.195	4.77	**<0.0001**	0.043
	CO	-0.002	0.003	-0.631	0.529	
	LVIV_d [500]	0.004	0.002	1.753	0.082	
	LVIV_s	-0.0002	0.0004	-0.509	0.612	
	SV	0.0001	0.0002	0.587	0.558	
Model 6 (great vessels)	Intercept	-0.094	0.562	-0.167	0.868	0.046
	AoD_{rlx-ed} [500]	0.141	0.075	1.893	0.060	
	PAD_{rlx-ed} [500]	0.069	0.054	1.301	0.195	
Model 7 (regurgitation severity, signalment, weight, creatinine, great vessels)	Intercept	-0.106	0.560	-0.188	0.851	0.187
	Regurgitation-Mild	-0.036	0.131	-0.274	0.784	
	Regurgitation-Moderate	0.012	0.135	0.087	0.9306	
	Regurgitation-Severe	-0.097	0.175	-0.554	0.580	
	Age	0.014	0.007	1.894	0.0602	
	Sex- FC	0.039	0.482	0.082	0.935	
	Sex- M	-0.182	0.119	-1.529	0.128	
	Sex-MC	-0.160	0.088	-1.813	0.072	
	Weight	-0.001	0.001	-1.525	0.129	
	Creatinine	0.008	0.002	4.007	**<0.0001**	
	AoD_{rlx-ed}	0.119	0.072	1.660	0.099	
	PAD_{rlx-ed}	0.053	0.055	0.966	0.335	
Model 8 (regurgitation severity,	Intercept	-0.529	0.695	-0.761	0.448	0.187

signalment, weight, creatinine, great vessels allometrically scaled)						
	Regurgitation-Mild	-0.036	0.131	-0.274	0.784	
	Regurgitation-Moderate	0.012	0.135	0.088	0.930	
	Regurgitation-Severe	-0.099	0.175	-0.568	0.571	
	Age	0.014	0.008	1.892	0.061	
	Sex- FC	0.041	0.482	0.086	0.932	
	Sex- M	-0.180	0.119	-1.518	0.131	
	Sex-MC	-0.160	0.088	-1.815	0.072	
	Weight	-0.001	0.001	-0.716	0.475	
	Creatinine	0.008	0.002	4.006	**<0.001**	
	AoD_{rlx-ed} [500]	0.124	0.075	1.655	0.100	
	PAD_{rlx-ed} [500]	0.053	0.056	0.950	0.344	
